# Thermal stability and structural changes in bacterial toxins responsible for food poisoning

**DOI:** 10.1371/journal.pone.0172445

**Published:** 2017-02-16

**Authors:** Paulina Regenthal, Jesper S. Hansen, Ingemar André, Karin Lindkvist-Petersson

**Affiliations:** 1 Department of Experimental Medical Science, Lund University, BMC, Lund, Sweden; 2 Department of Biochemistry and Structural Biology, Lund University, Lund, Sweden; Russian Academy of Medical Sciences, RUSSIAN FEDERATION

## Abstract

The staphylococcal enterotoxins (SEs) are secreted by the bacteria *Staphylococcus aureus* and are the most common causative agent in staphylococcal food poisoning. The staphylococcal enterotoxin A (SEA) has been associated with large staphylococcal food poisoning outbreaks, but newer identified SEs, like staphylococcal enterotoxin H (SEH) has recently been shown to be present at similar levels as SEA in food poisoning outbreaks. Thus, we set out to investigate the thermo-stability of the three-dimensional structures of SEA, SEH and staphylococcal enterotoxin E (SEE), since heat inactivation is a common method to inactivate toxins during food processing. Interestingly, the investigated toxins behaved distinctly different upon heating. SEA and SEE were more stable at slightly acidic pH values, while SEH adopted an extremely stable structure at neutral pH, with almost no effects on secondary structural elements upon heating to 95°C, and with reversible formation of tertiary structure upon subsequent cooling to room temperature. Taken together, the data suggests that the family of staphylococcal enterotoxins have different ability to withstand heat, and thus the exact profile of heat inactivation for all SEs causing food poisoning needs to be considered to improve food safety.

## Introduction

The bacterium *Staphylococcus aureus* secretes a wide variety in virulence factors like the staphylococcal superantigen [1]. These superantigens (SAgs) include the staphylococcal enterotoxins (SEs), the staphylococcal enterotoxin-*like* (SE*l*s) proteins, and toxic shock syndrome toxin-1 (TSST-1). The SAgs bind directly to MHC class II molecules on antigen presenting cells and to the T cell receptor on T cells and stimulate a large population of T cells, which may result in disease [2]. The SEs are recognized as common causative agents of human staphylococcal food poisoning (SFP), which stems from the consumption of foods containing sufficient amounts of preformed SEs [3]. The SEs are generally known to be highly heat resistant and tolerate low pHs [4]. Still, food is commonly thermally processed at acidic pH, which could then lead to inefficient neutralization of potential enterotoxins. Staphylococcal enterotoxin A (SEA) is reported as the most frequent enterotoxin involved in SFP, responsible for approximately 80% of the outbreaks in the US [5]. Other staphylococcal enterotoxins (SED, SEE and SEH) are also associated with food-poisoning world-wide, but possibly less frequently than SEA [5]. Thermal processing is one of the most useful tools to effectively reduce the amounts of bacteria that may potentially be present in meat, poultry and dairy products. The bacteria will in most cases be inactivated during this process, but preformed toxins such as the SEs, may persist and cause SFP in the future consumer.

The SEA, SEE and SEH toxins share a common three-dimensional fold and biological activity. Still, specific SEs have more frequently than others been identified in SFP outbreaks in certain types of foods. For instance, SEH has commonly been detected in food-poisoning outbreaks caused by dairy products such as milk and cheese [6, 7], while SEA and SEE have been detected in a broader manner in meat, poultry, dairy and vegetables [8]. Although, SFP has been studied to great extent there is a lack of detailed understanding of the mechanism behind heat inactivation and reactivation at lower temperature of different SEs. The superantigens have been divided into five evolutionary groups (I-V) [9] based on sequence similarity, and SEA belongs to the evolutionary group III. Toxins belonging to group III are able to cross-link two MHC class II molecules through one low-affinity binding site [10] and another zinc-dependent high-affinity binding site [11]. Within group III, SEA has the highest sequence identity with SEE (82%) and lowest with SEH (31%), based on sequence alignments with CLUSTAL W [12]. To investigate how the three-dimensional structures of SEA, SEE and SEH may be affected during food preparation, the changes in secondary and tertiary structure were investigated at different pH values (5–7) upon heating. Since the group III SEs have a zinc site located to the C-terminal domain (essential for MHC class II binding and T cell activation), we investigated the three-dimensional structures in the presence of either zinc or EDTA to clarify any putative role of the zinc ion. The presence of zinc has previously been suggested to thermally stabilize SEA and SEE [13]. In this study, we applied a combination of CD and fluorescence spectroscopy to characterize the protein structures at the secondary and at the tertiary structural level. Interestingly, the investigated toxins, all known to have emetic effects and contribute to SFP, behaved different upon heating. Firstly, SEH was extremely stable at neutral pH, with no observed changes in secondary structure and reversible changes at the tertiary level upon heating, while SEA and SEE aggregated, followed by visual precipitation at neutral pH. Secondly, at acidic pH in the presence of zinc, SEA and SEE gained secondary structure upon heating, and possible adopted an alternatively folded state accompanied by exposure of hydrophobic elements. This clearly suggests that for sufficient heat inactivation of SE-containing foods, it is important to identify which SEs that are present and determine the exact mechanism of heat inactivation and reactivation for those specific SEs. In this manner, safer guidelines for food preparation and conservation may be established in the effort to improve food quality and safety.

## Materials and methods

### Protein expression and purification

SEA and SEH were expressed and purified as described previously with some modifications [14]. Two vectors (gifts from Active Biotech Research AB, Lund, Sweden) containing the constitutive promotor A and a gene for kanamycin resistance, one with the SEA gene and the other one with the SEH gene, were each transformed into *Escherichia coli* K12 strain UL635 for expression of SEA and SEH. The culture medium used was 2xYT media (16 g/L tryptone, 10 g/L bacto yeast extract, 5g/L NaCl) supplemented with 100 mg/L kanamycin, 90 mM Potassium Phosphate pH 7.0, 0.5% glycerol, 0.1% trace elements and 0.5% glucose, and the proteins were constitutively expressed at 26–29°C for 17 h. The cells were harvested by centrifugation at 8000 g at 4°C for 15 min. Periplasmic protein content was extracted by hypotonic shock by resuspending the cells into 45 mM Tris-HCl, pH 7.5 supplemented with 1 mM EDTA. Following equilibration on ice for 1 h with agitation, the cell extract was cleared by centrifugation at 4000 g at 4°C for 1 h. The cleared supernatant was adjusted to pH 5.7 for SEA and pH 4.5 for SEH. Both supernatants were centrifuged to remove possible precipitation (4000 g, 40 min, 4°C). The SEA sample was diluted 13 times in 10 mM potassium phosphate pH 5.8 and SEH was diluted 10 times in 20 mM ammonium acetate pH 4.2. Both protein solutions were filtered through a 0.2 μm filter and loaded onto a 6 ml Resource S column (GE Healthcare) for purification using cation exchange chromatography. SEA was purified with a linear gradient using 500 mM NaCl in 10 mM potassium phosphate pH 5.8 supplemented with 0.05% v/v Tween-20, while the elution buffer for SEH consisted of 500 mM ammonium acetate pH 4.5 supplemented with 0.05% v/v Tween-20. The proteins were further purified by size-exclusion chromatography (SEC). SEA was purified using first a HiLoad Superdex 200 16/60 GL column (GE Healthcare) followed by a Superdex 75 10/300 GL column (GE Healthcare) and SEH using only the Superdex 75 10/300 GL column, using 20 mM NaH_2_PO_4_ pH 6.0 as running buffer. Purified SEE was a kind gift from Active Biotech Research AB (Lund, Sweden), which was expressed and purified according to published protocols [15]. Before use, SEE was subjected to size-exclusion chromatography (Superdex 75 10/300 GL column, GE Healthcare) also using 20 mM NaH_2_PO_4_ pH 6.0 as running buffer.

### Circular Dichroism (CD) spectroscopy

Far-UV CD spectra and thermal denaturation were recorded using a Jasco J-810 spectropolarimeter equipped with a Peltier thermo-stated cell holder. A 1 mm quartz cuvette was used, and thermo stated at 20°C, unless otherwise stated. The measurement range was 195–260 nm, and spectra were recorded at a scan rate of 20 nm/min with a 0.1 nm data pitch, a response time of 0.25 sec, and with 8 accumulations. All CD spectra were background subtracted using the appropriate buffer (20 mM NaH_2_PO_4_) at indicated pH values without protein. The thermal denaturation measurements were recorded at 220 nm with 1°C increments and a response time of 4 sec. The temperature was ramped from 40 to 95°C with a rate of 1°C/min. Results were obtained in millidegrees (mdeg) and subsequently converted to the mean residue ellipticity (MRE) ([θ] × mdeg cm^2^ dmol^−1^) based on 233 amino acid residues and a molecular weight of 27.1 kDa for SEA and 26.8 kDa for SEE, respectively, and 217 amino acids and a molecular weight of 25.1 kDa for SEH. Protein concentrations used were 0.2 mg/ml, determined by UV/Vis absorbance spectroscopy using the extinction coefficient at 280 nm of 37945 M^-1^ cm^-1^ for SEA, 36455 M^-1^ cm^-1^ for SEE and 26485 M^-1^ cm^-1^ for SEH. Deconvolution of the CD spectra into pure component spectra was performed using the CD secondary structure analysis algorithm BeStSEl with the data range from 200 to 250 nm [16]. The thermal denaturation measurements were plotted, the data were fitted using non-linear regression and the transition temperatures (T_s_) were determined in GraphPad Prism 6.

### SDS-PAGE

SEA, SEE and SEH (0.1 mg/ml) were subjected to consecutive heating using a standard heat block. The temperature was ramped from 50 to 95°C, the increments were 5°C and equilibration time was 5 min. Following thermal challenge of proteins at the specified temperature intervals, an aliquot of each protein was placed on ice, transferred to a new Eppendorf tube and diluted into LDS sample buffer 4x (Invitrogen, USA) containing 0.2 M dithiothreitol (DTT). The protein samples (1 μg protein) were loaded together with a PageRuler prestained protein ladder (Thermo Scientific) on separate 4–12% Bis-Tris gels for SDS-PAGE. Protein bands were stained with Simply Blue Safe Stain (Invitrogen) and all proteins were determined to at least 95% purity. The amount of the consecutive heated proteins that entered the gels was quantified using the gel analysis tool in ImageJ [17].

### Fluorescence spectroscopy

Fluorescence spectra were recorded using a Jasco J-810 spectropolarimeter equipped with a FMO-427S fluorescence module. A 10 mm × 10 mm quartz cuvette was used. The excitation wavelength was 280 nm, and emission was recorded between 300 and 420 nm. Spectra were recorded with 5°C increments and 1 min equilibrations between the temperature range from 20 to 95°C. The protein concentration was 25 μg/ml in 20 mM NaH_2_PO_4,_ pH values as indicated. The background of the buffers alone without protein was subtracted from all protein fluorescence spectra and baselines were adjusted.

## Results and discussion

### The superantigens SEA, SEE and SEH were produced to homogeneity

**Fig 1:**
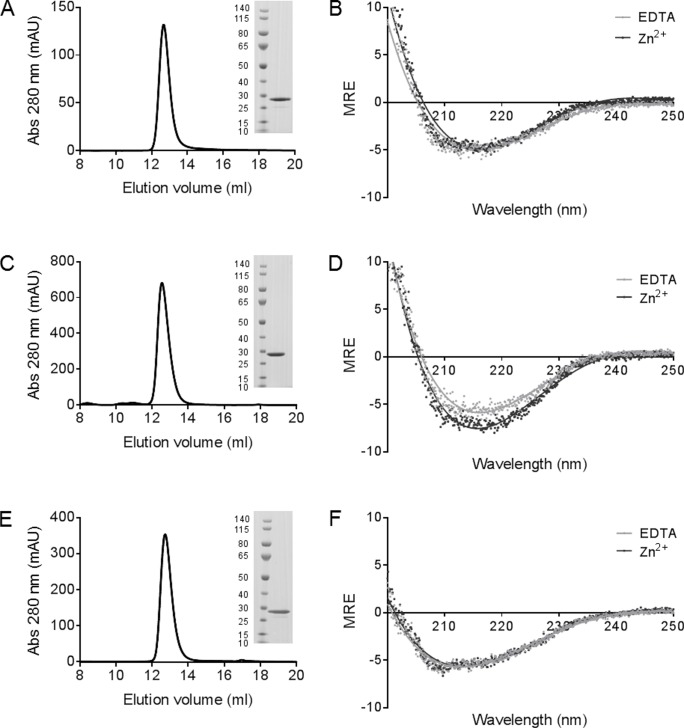
Purification of natively folded SEA, SEE and SEH. (A, C, E) Size-exclusion chromatography of SEA, SEE and SEH, with protein purities analyzed by coomassie-stained SDS-PAGE gels shown as insets. (B, D, F) Far-UV CD spectra for SEA, SEE, SEH at 200–250 nm at 20°C in presence of 0.1 mM ZnCl_2_ (dark grey) or 1 mM EDTA (light grey) at pH 6.0.

Superantigens are known to have a very stable structure at room temperature. To clarify the exact structural mechanisms of heat inactivation and/or reactivation of bacterial toxins causing food poisoning, SEA, SEE and SEH were produced in mg quantities (Fig 1). SEA and SEH were constitutively expressed in *E*. *coli* using promoter A and purified applying ion-exchange chromatography. The yield was approximately 1 mg protein/l culture of all produced toxins. All proteins adopted a proper three-dimensional fold judged by size-exclusion chromatography, and the purity was estimated by SDS-PAGE to be at least 95% for each protein (Fig 1A, 1C and 1E). To analyze the secondary structure of the toxins, far-UV CD spectra were recorded in the wavelength range from 195 and 260 nm, in presence of Zn^2+^ or EDTA (Fig 1B, 1D and 1F). The secondary structural content of each toxin was estimated using the CD secondary structure analysis tool BeStSEl [16]. The overall results demonstrate a good correlation between the experimentally determined secondary structure contents of SEA, SEE and SEH, and the secondary structure estimated from the known three-dimensional structures of the proteins, with a somewhat lower value for the α-helical content possible due to that fact that it is more difficult to deconvolute secondary structure in proteins that contain large amount of β-sheets (Table 1) [18–20].

**Table 1:** Theoretical and experimental content of secondary structure (%) determined for SEA, SEE and SEH at pH 6.0 in presence of EDTA or Zn^2+^.

SE protein	α-helix	β-sheet	Turn	Unordered	RMSD**
Theoretical SEA (1ESF)*	17.6	32.1	12.0	38.3	−
SEA pH 6.0 EDTA	10.5	39.4	16.5	33.6	0.119
SEA pH 6.0 Zn^2+^	12.2	41.7	9.3	36.8	0.101
Theoretical SEE (5FK9)*	15.5	26.5	8.2	49.8	−
SEE pH 6.0 EDTA	6.6	46.2	14.4	32.8	0.151
SEE pH 6.0 Zn^2+^	8.6	32.6	39.9	39.9	0.178
Theoretical SEH (1ENF)*	18.3	41.0	9.0	31.7	−
SEH pH 6.0 EDTA	13.2	37.4	11.2	38.2	0.093
SEH pH 6.0 Zn^2+^	12.1	31.1	12.5	44.3	0.084

*PDB accession code

**RMSD between experimental and fitted data.

### Heat has different impact on the secondary structures of SEA, SEE and SEH

**Fig 2:**
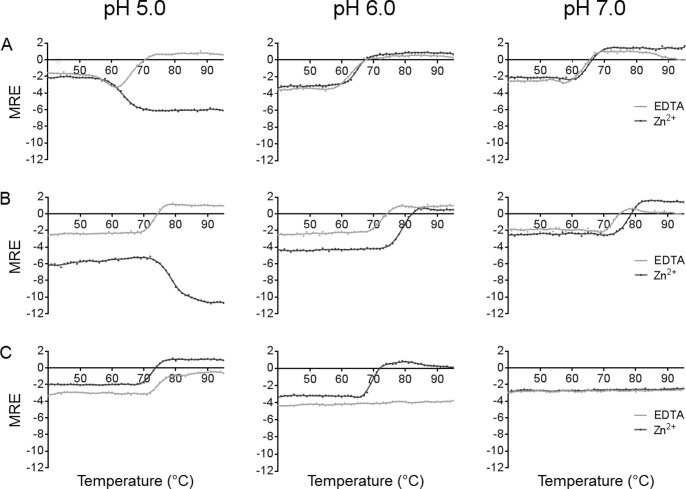
Thermal denaturation of SEA, SEE and SEH at different pH values. Transition curves upon heating for (A) SEA, (B) SEE and (C) SEH in presence of 0.1 mM ZnCl_2_ (dark grey) or 1 mM EDTA (light grey) measured by CD spectroscopy at 220 nm, from 40–95°C.

The manufacturing process of food is commonly using heat treatment as a major inactivation step for SEs, and hence we set out to clarify the impact heating has on the secondary structural elements of SEA, SEE and SEH at pH 5.0, 6.0 and 7.0 by application of CD spectroscopy. At pH 5.0, SEA was prone to aggregate upon heating in presence of EDTA, judged by loss of detectable signal after the transition (S1A Fig) and detection of visual precipitation after heating. SEE behaved similarly but with a transition temperature ~6°C higher than SEA, which correlates well to previously published data (Fig 2, Table 2) [13]. At pH 5.0, in the presence of Zn^2+^, a decrease in ellipticity was detected for both SEA and SEE upon heating (Fig 2A and 2B, S1B Fig). This can be interpreted as a change in secondary structure, primarily in alpha-helical content and indicates that SEA and SEE adopt a distinct fold from the native state upon heating. Alternatively, this can also result from a change in secondary structure type from β to α in segments of the protein. For SEH at pH 5.0, the negative ellipticity of the protein was lost at higher temperature, both in presence of EDTA and in presence of Zn^2+^ at temperatures from 75°C and above, with thermal transitions comparable to SEE (in presence of EDTA) (Fig 2, Table 2). At pH 6.0 and 7.0, SEA and SEE showed a positive ellipticity upon heating independently of the presence of EDTA or Zn^2+^, with SEE having consistently higher transition temperatures than SEA (Fig 2A and 2B, Table 2). SEH on the other hand behaved distinctly different. At pH 6.0, the presence of Zn^2+^ caused changes in secondary structure of SEH, but in the presence of EDTA, the secondary structure was unaffected by increases in temperature (Fig 2C). At pH 7.0, this was even more pronounced for SEH, and independently of the presence of Zn^2+^ or EDTA, the secondary structure (as quantified by mean residue ellipticity) was not changed upon heating (Fig 2C). Hence, SEH has an extremely stable secondary structure at neutral pH, and may not be thermally inactivated under such conditions. This correlates well with that SEH has a theoretical isoelectric point (pI) at 5.2, while SEA and SEE have pI at 6.6, suggesting that SEH would be less prone to aggregate in pH 7, while SEA and SEE would be less prone to aggregate at pH 5.

**Table 2:** Transition temperatures (T_s_ values, [°C]) of SE secondary structures at pH 5.0–7.0 in presence of EDTA or Zn^2+^.

	pH 5.0		pH 6.0		pH 7.0	
**SE protein**	**EDTA**	**Zn**^**2+**^	**EDTA**	**Zn**^**2+**^	**EDTA**	**Zn**^**2+**^
SEA	66.8	63.8	62.9	64.8	63.6	65.3
SEE	73.3	78.6	72.7	79.0	73.1	77.8
SEH	74.5	72.6	ND	69.4	ND	ND

### The effects on the tertiary structure upon heating and cooling

**Fig 3:**
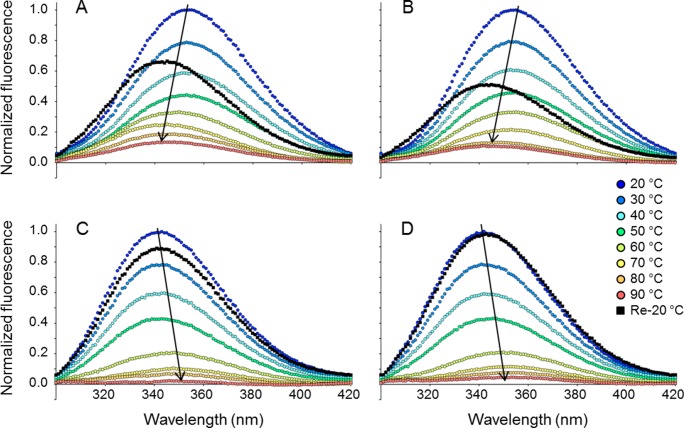
Intrinsic fluorescence for SEA, SEE and SEH as a function of temperature. Thermal tertiary structure changes of SEA, SEE and SEH were monitored by tryptophan/tyrosine fluorescence. (A) SEA in the presence of 0.1 mM ZnCl_2_ at pH 5.0, (B) SEE in the presence of 0.1 mM ZnCl_2_ at pH 5.0, (C) SEH at pH 7.0 in the presence of 0.1 mM ZnCl_2_ or (D) SEH at pH 7.0 in the presence of 1 mM EDTA. The excitation wavelength was 280 nm. Temperatures are represented by colors from blue, 20°C to red, 90°C. Black arrows indicate fluorescence emission maxima red- or blue-shifting.

**Fig 4:**
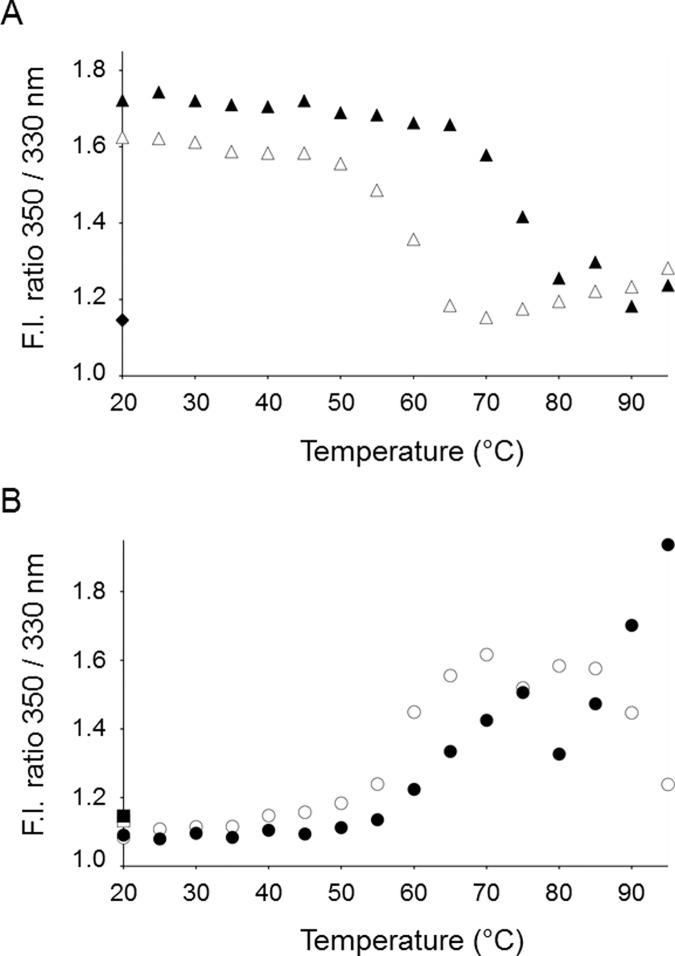
Fluorescence emission maxima changes for SEA, SEE and SEH as a function of temperature. Changes at the tertiary structure level upon heating of SEA, SEE and SEH were monitored as the fluorescence intensity ratio 350/330 nm. The data were derived from the fluorescence spectra shown in Fig 3. (A) SEA and SEE at pH 5.0 in the presence of 0.1 mM ZnCl_2_. The data points for SEA are shown as white triangles (Δ), while SEE data points are depicted as black triangles (▲). Furthermore, SEA after re-cooling (◊) and SEE after re-cooling (♦) are shown. (B) SEH at pH 7.0 in the presence of 1 mM EDTA (○) or 0.1 mM ZnCl_2_ (●). In addition, SEH after re-cooling in presence of 1 mM EDTA (□) or presence of 0.1 mM ZnCl_2_ (■) are shown.

In most conditions evaluated for thermal denaturation of secondary structure, SEA, SEE and SEH did in fact aggregate, followed by visual precipitation. However, there are four particularly interesting conditions where the secondary structure was either unaffected by the heating (Fig 2C) or even increased (Fig 2A and 2B). To investigate how the tertiary structure is altered upon heating in these specific conditions, the intrinsic fluorescence of the proteins was assessed by fluorescence spectroscopy (Fig 3). The emission wavelengths of tryptophans and tyrosines are strongly dependent on their immediate environment surrounding the aromatic side chains [21]. Thus, a change in tertiary structure can be detected if it leads to a change of polarity around the aromatic residues. SEA and SEE have two tryptophan residues at positions 63 and 130 (SEA numbering), whereas SEH only has the tryptophan corresponding to residue 130. According to the X-ray structures, their common tryptophan is situated near the zinc binding site in the C-terminal domain [15, 18, 19], whereas the extra tryptophan in SEA and SEE is situated in a loop in the N-terminal domain at the opposite side of the protein. In addition, all the investigated SEs have several tyrosine residues. Thus, the fluorescence emission of these proteins upon excitation can be used to investigate changes in tertiary structure upon heating and cooling. Emission spectra in the range of 300 to 420 nm were recorded by exciting both tryptophans and tyrosines at 280 nm. Since SEA and SEE showed as a change in secondary structure, indicating that they adopt a distinct fold from the native state upon heating at pH 5.0 in presence of Zn^2+^ (Fig 2A and 2B), changes in intrinsic fluorescence upon heating and cooling were measured for SEA and SEE at this condition. At room temperature, SEA and SEE have fluorescence emission spectra with maxima around 350 nm, indicating that the spectra are dominated by fully exposed Trp/Tyr side chains, which is in accordance with previously published data (Fig 3A and 3B) [22]. Upon thermal increase, the Tyr/Trp fluorescence emission maxima showed a clear blue-shift, as evidenced by a change in the fluorescence intensity ratio 350/330 nm (Fig 4A). This blue-shift correlated well with the transition curves obtained with CD spectroscopy under these conditions (Figs 2 and 4A). The Tyr/Trp fluorescence revealed that the apparent increase in secondary structure of the proteins upon heating is associated with a concomitant decrease in polarity surrounding the aromatic side chains of SEA and SEE. In contrast, SEH measured at pH 7.0, where the secondary structure is unaffected by heating, exhibited fluorescence emission maxima around 340 nm with both EDTA and Zn^2+^, indicating that the Tyr/Trp in SEH are exposed to bound water possessing very long dipole relaxation time [21]. Thus, the aromatic residues are less solvated in the native state of SEH initially compared to both SEA and SEE. While the secondary structure of SEH was unaffected by heating, a clear red-shift, resulting from an increase in polarity around the aromatic residues, was observed (Fig 3C and 3D). Plotting the 350/330 nm fluorescence intensity ratio with increasing temperature, revealed that SEH changed its conformation at the tertiary structure level at pH 7.0, in the presence of EDTA, around 60°C, whereas the presence of Zn^2+^ increased the thermal stability of SEH by approximately 10°C (Fig 4B). The fluctuations in the 350/330 values observed above 80°C for SEH in presence of EDTA or Zn^2+^, reflect loss of fluorescence signal as a consequence of increase in temperature and thus complete solvent exposure of aromatic residues, rather than altered polarity per se (Fig 4B). Interestingly, when SEH was cooled to the initial temperature of 20°C, the fluorescence emission maxima returned to its initial emission maxima of 340nm. Thus, the tertiary structural changes of SEH, as observed by fluorescence due to heating, are reversible during cooling, while this is not the case for SEA and SEE (Figs 3 and 4). This suggests that all the investigated proteins adopt a conformational state different from the native state upon heating. SEA and SEE possibly form an alternative fold with exposed hydrophobic elements upon heating, while SEH adopts changes in tertiary structure conformation that are reversible upon cooling.

### Consecutive thermal increase induces oligomerization of SEA and SEE

**Fig 5:**
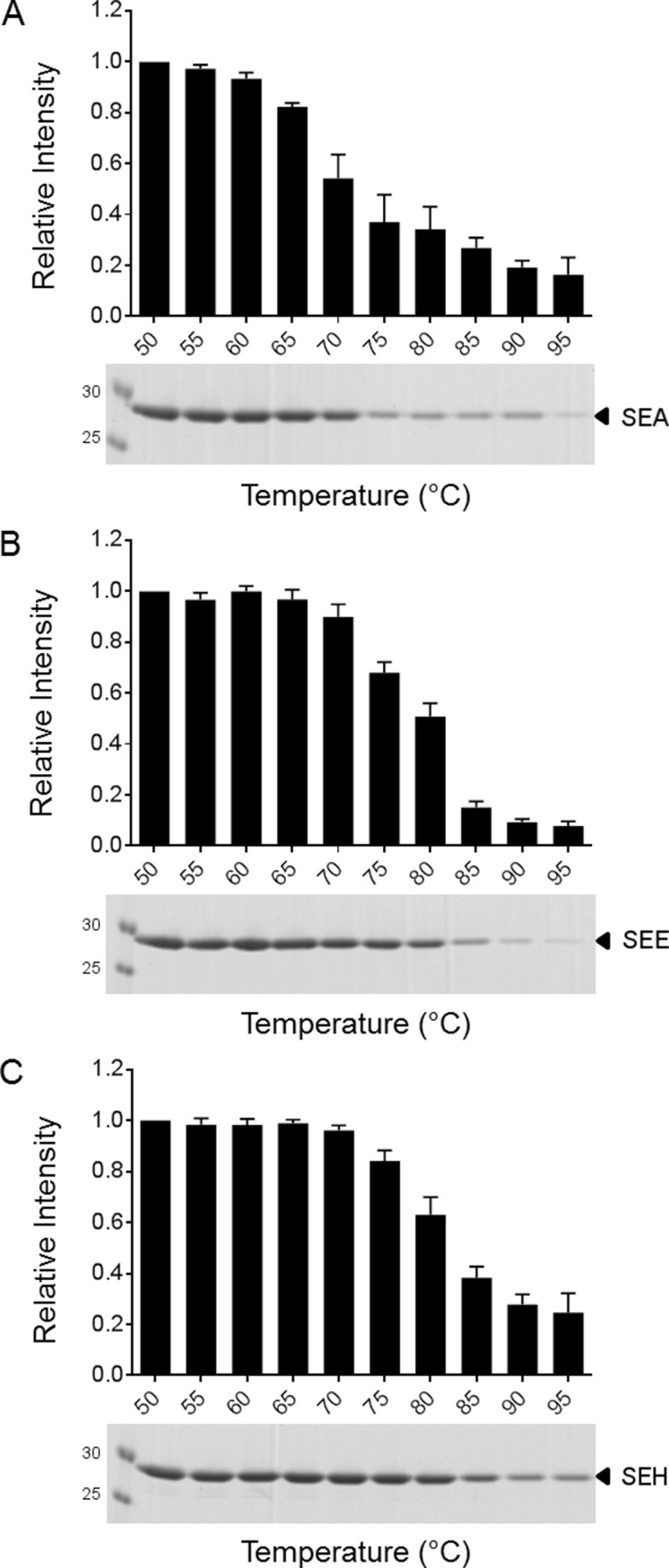
Consecutive heating of SEA, SEE and SEH. (A) SEA at pH 5.0, (B), SEE at pH 5.0 and (C) SEH at pH 7.0, all in the presence of 0.1 mM ZnCl_2_, were consecutively heated from 50°C to 95°C with 5°C increments. Each protein was subjected to the indicated temperature for 5 min, and then cooled, before applied to SDS-PAGE. The amount of protein entering the gel was quantified by ImageJ and normalized to the protein level at the starting point (50°C). Error bars are the means ± SEM of four independent experiments. Representative coomassie-stained SDS-PAGE gels are shown.

To clarify whether the blue-shift of SEA and SEE, detected using fluorescence spectroscopy upon heating, is a result of oligomerization, the toxins were exposed to consecutive heating within the temperature interval 50–95°C and analyzed using sodium dodecyl sulfate polyacrylamide gel electrophoresis (SDS-PAGE) (Fig 5). The consecutive heating was performed in 5°C increments with 5 min equilibration time at each step to ensure thermal increase of the protein samples. This is similar to the temperature increase the proteins were exposed to in the CD and fluorescence spectroscopy experiments, only with prolonged incubation times. If large oligomers are formed upon heating, these will fail to penetrate the stacking gel in the SDS-PAGE, enabling evaluation of the extent of multimeric formation [23]. As seen in Fig 5, when heated above 85°C, SEA and SEE can no longer enter the gel (Fig 5A and 5B). In contrast, SEH still partly enters the gel even after exposure to 95°C (Fig 5C). This suggests that SEA and SEE seem to oligomerize upon heating and subsequent cooling, while SEH seems to be less affected and can possibly fold back to its native structure upon cooling, as also seen in the fluorescence spectroscopy (Fig 4B). The partial failure to refold and hence to enter the stacking gel for SEH compared to the fluorescence spectroscopy data, could be explained by the longer thermal exposure time compared to both CD and fluorescence spectroscopy methods.

### The secondary structure is still present after heating and subsequent cooling for SEA and SEE

**Fig 6:**
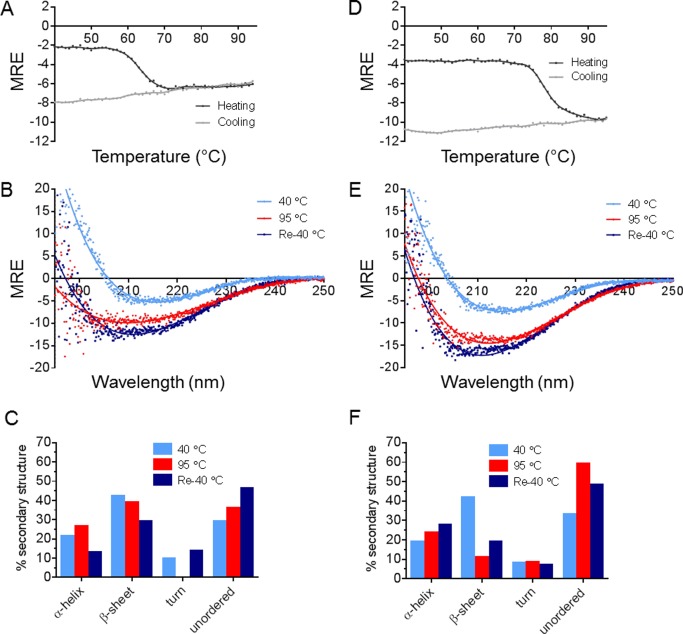
Evaluation of secondary structure for SEA and SEE after heating and re-cooling. Transition curves upon heating (40°C—95°C, dark grey) and cooling (40°C, light grey) were recorded by CD spectroscopy at 220 nm for (A) SEA and (D) SEE at pH 5.0 in presence of 0.1 mM ZnCl_2_. Far-UV CD spectra (195–250 nm) for (B) SEA and (E) SEE at pH 5.0 in presence of 0.1 mM ZnCl_2_ recorded at 40°C (blue), 95°C (red), and after subsequent cooling to 40°C (dark blue). Quantification of secondary structural elements using the far-UV CD spectra for (C) SEA and (F) SEE at 40°C (blue), upon heating to 95°C (red) and cooling back to 40°C (dark blue). The RMSD between experimental and fitted data for SEA is: 0.235, 0.615 and 0.152 for initial 40°C, 95°C and after subsequent cooling to 40°C, respectively. The RMSD values for SEE are 0.157, 0.179 and 0.153, respectively, for the same conditions.

The increase in non-polar environment for the Tyr/Trp residues detected upon heating in fluorescence spectroscopy, and the failure to enter the SDS-PAGE gel upon heating, suggest that SEA and SEE may partially denature upon heating and form larger oligomers at this condition. To clarify if this is simply a process accompanied by loss of secondary and tertiary structure, the thermal denaturation profiles on secondary structure level for SEA and SEE were monitored applying CD spectroscopy for subsequent Peltier element controlled heating and cooling (Fig 6A and 6D). Far-UV CD spectra were recorded at all start and end thermal measurement points (Fig 6B and 6E). As seen in Fig 6, SEA and SEE have significant degree of secondary structure at 95°C as well as after cooling the samples to 40°C (Fig 6B and 6E). Furthermore, the conformational change triggered by heat is not reversible upon cooling with the heat-treated proteins having distinctly lower ellipticity at 220 nm than the starting material (Fig 6A and 6D). To investigate the content of the secondary structure elements in SEA and SEE at 40°C, 95°C and after subsequent cooling to 40°C, the CD secondary structure analysis tool BeStSEl was used [16]. The minima shift towards lower wavelengths observed for both SEA and SEE upon heating and re-cooling is consistent with an increase in the amount of unordered structure suggested by the BeStSEl analyses (Fig 6). In addition, the BeStSEl analyses show a small increase in the amount of α-helices for SEA and SEE upon heating. This suggests that although the secondary structure elements are still present after the thermal processing, the overall structure of the proteins are affected, which correlates well with the fluorescence data. Thus, at this condition both SEA and SEE possibly adopt an alternative folded state different from both the native and the aggregated state.

## Conclusion

Some SEs clearly aggregate upon heating, while other can persist as biologically intact molecules and adopt non-native structural conformations that may be reversible upon cooling. Here we show that SEA and SEE aggregate upon heating in most conditions, which correlates well with previously published data [13]. However, at pH 5.0 in the presence of Zn^2+^, secondary structure elements persisted heating for both SEA and SEE and hydrophobic elements buried in the native fold, were exposed to the environment, indicating formation of an alternatively folded state. Interestingly, partially unfolded SEA has previously been detected to have an increased T cell activity [22]. Meat, poultry and vegetables commonly have a pH below 6, and since SEA is the most common cause of SFP an increase in pH may be advantageous during thermal processing of food to ascertain a full destruction, at both the secondary and the tertiary structural level, for SEA and SEE. The SEH superantigen, on the other hand, adopted an aggregated state, followed by precipitation upon thermal increase at pH 5.0, but at neutral pH the secondary structure was completely unaffected by any increase in temperature. At the tertiary structural level, the toxin did unfold, but upon cooling it re-gained its tertiary structure. Interestingly, SEH has commonly been associated with SFP outbreaks caused by dairy products, which has neutral pH. Hence, our data supports that SEH could retain its three-dimensional structure during thermal processing of dairy products at neutral pH.

## Supporting information

S1 Fig Full far-UV CD spectra collected upon heating for SEA. Far-UV CD spectra recorded on SEA at pH 5.0 in the presence of (**A**) 1 mM EDTA (**B**) or 0.1 mM ZnCl_2_. The spectra were recorded between 195–260 nm, with 2°C increments and a temperature ramp from 50 to 80°C at a rate of 1°C/min. Ellipticity [CD] for each spectrum measured in millidegrees (mdeg). Protein concentrations used were 0.2 mg/ml in 20 mM NaH_2_PO_4_ at pH 5.0 supplemented with either 1 mM EDTA or 0.1 mM ZnCl_2_.
(DOCX)
