# Correction: Thermal stability and structural changes in bacterial toxins responsible for food poisoning

**DOI:** 10.1371/journal.pone.0175989

**Published:** 2017-04-12

**Authors:** Paulina Regenthal, Jesper S. Hansen, Ingemar André, Karin Lindkvist-Petersson

There is an error with the Turn content of the SEE pH 6.0 Zn^2+^ sample. Please see the corrected Table 1 here.

**Table 1:** Theoretical and experimental content of secondary structure (%) determined for SEA, SEE and SEH at pH 6.0 in presence of EDTA or Zn^2+^.

SE protein	α-helix	β-sheet	Turn	Unordered	RMSD**
Theoretical SEA (1ESF)*	17.6	32.1	12.0	38.3	−
SEA pH 6.0 EDTA	10.5	39.4	16.5	33.6	0.119
SEA pH 6.0 Zn^2+^	12.2	41.7	9.3	36.8	0.101
Theoretical SEE (5FK9)*	15.5	26.5	8.2	49.8	−
SEE pH 6.0 EDTA	6.6	46.2	14.4	32.8	0.151
SEE pH 6.0 Zn^2+^	8.6	32.6	18.9	39.9	0.178
Theoretical SEH (1ENF)*	18.3	41.0	9.0	31.7	−
SEH pH 6.0 EDTA	13.2	37.4	11.2	38.2	0.093
SEH pH 6.0 Zn^2+^	12.1	31.1	12.5	44.3	0.084

*PDB accession code,

**RMSD between experimental and fitted data.
