# Supplementary material for: Thermal stability and structural changes in bacterial toxins responsible for food poisoning
Source: PLoS One. 2017 Feb 16;12(2):e0172445. doi: 10.1371/journal.pone.0172445 (PMC5313198; doi:10.1371/journal.pone.0172445)
Supplement: S1 Fig — Far-UV CD spectra recorded on SEA at pH 5.0 in the presence of (A) 1 mM EDTA (B) or 0.1 mM ZnCl2. The spectra were recorded between 195–260 nm, with 2°C increments and a temperature ramp from 50 to 80°C at a rate of 1°C/min. Ellipticity [CD] for each spectrum measured in millidegrees (mdeg). Protein concentrations used were 0.2 mg/ml in 20 mM NaH2PO4 at pH 5.0 supplemented with either 1 mM EDTA or 0.1 mM ZnCl2. (DOCX) [file pone.0172445.s001.docx]

**Supporting Information for**

**Thermal stability and structural changes in bacterial toxins responsible for food poisoning**

*Short title: Thermal stability of staphylococcal enterotoxins*

Paulina Regenthal^1^, Jesper S Hansen^1^, Ingmar Andre^2^, Karin Lindkvist-Petersson^1^

1. Department of Experimental Medical Science, Lund University, BMC, 221 84, Lund, Sweden.
2. Department of Biochemistry and Structural Biology, Lund, University, PO Box 124, SE-221 00, Lund, Sweden.

**Supporting Figures**

**
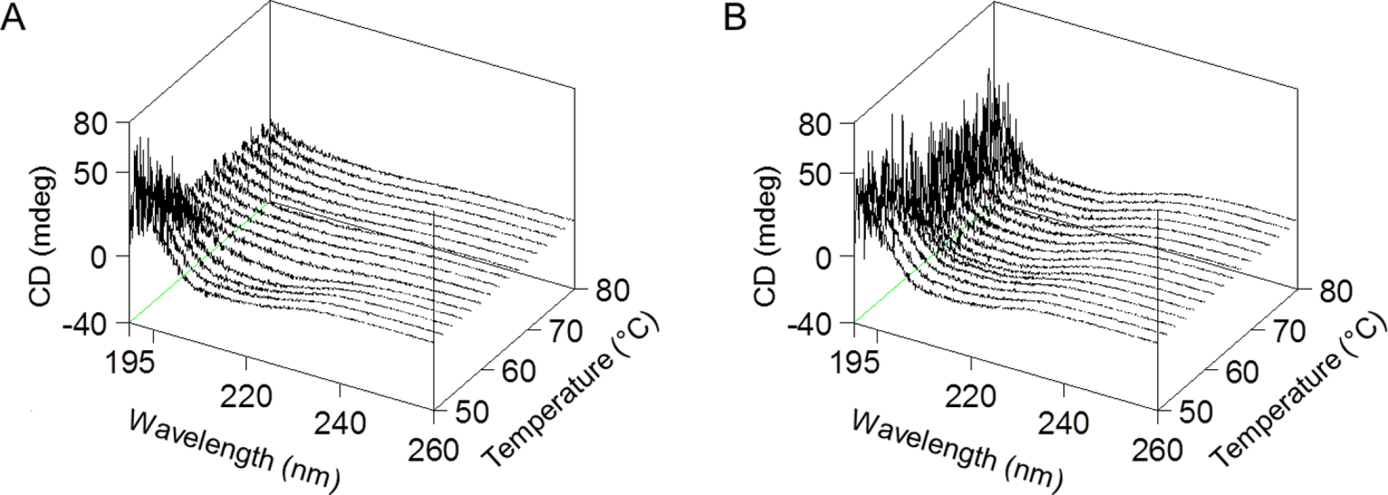
**

**S1 Fig. Full far-UV CD spectra collected upon heating for SEA.** Far-UV CD spectra recorded on SEA at pH 5.0 in the presence of (**A**) 1 mM EDTA (**B**) or 0.1 mM ZnCl_2_. The spectra were recorded between 195-260 nm, with 2 ºC increments and a temperature ramp from 50 to 80 ºC at a rate of 1 ºC/min. Ellipticity [CD] for each spectrum measured in millidegrees (mdeg). Protein concentrations used were 0.2 mg/ml in 20 mM NaH_2_PO_4_ at pH 5.0 supplemented with either 1 mM EDTA or 0.1 mM ZnCl_2_.
